# Radiosensitization by Docetaxel Prodrug-Loaded Lipid Nanoparticles in Pancreatic Cancer Xenografts

**DOI:** 10.3390/nano15191521

**Published:** 2025-10-05

**Authors:** Abdulaziz Alhussan, Nolan Jackson, Nancy Dos Santos, Sam Chen, Yuen Yi C. Tam, Devika B. Chithrani

**Affiliations:** 1 Department of Biochemistry and Molecular Biology, University of British Columbia, Vancouver, BC V6T 1Z4, Canada; 2 Department of Physics and Astronomy, University of Victoria, Victoria, BC V8P 5C2, Canada; nolanjackson12@uvic.ca (N.J.); devikac@uvic.ca (D.B.C.); 3 Department of Experimental Therapeutics, British Columbia Cancer-Vancouver, Vancouver, BC V5Z IL3, Canada; ndossantos@bccrc.ca; 4 Integrated Nanotherapeutics Inc., Burnaby, BC V5G 4X4, Canada; samchen@integratedntx.com (S.C.); christam@integratedntx.com (Y.Y.C.T.); 5 Centre for Advanced Materials and Related Technologies, Department of Chemistry, University of Victoria, Victoria, BC V8P 5C2, Canada; 6 Department of Medical Sciences, University of Victoria, Victoria, BC V8P 5C2, Canada; 7 Department of Computer Science, Mathematics, Physics and Statistics, Okanagan Campus, University of British Columbia, Kelowna, BC V1V 1V7, Canada

**Keywords:** docetaxel, lipid nanoparticles, radiotherapy, cancer, docetaxel prodrug, radiosensitization, nanotechnology, combined modality therapy, gold nanoparticles

## Abstract

Cancer treatments are limited by poor tumor specificity and toxicity. We tested a radiosensitizing approach using PEG/RGD-functionalized gold nanoparticles (GNPs), a lipid-nanoparticle–encapsulated docetaxel prodrug (LNP_DTX–P_), and external-beam radiotherapy (RT). In MIA PaCa-2 xenografts, intravenous GNPs (2 mg/kg) and LNP_DTX–P_ (6 mg/kg) were given before 5 Gy RT. Both LNP_DTX–P_ + RT and GNPs + LNP_DTX–P_ + RT reduced tumor volume by ~40% and significantly prolonged survival versus RT alone (*p* < 0.001). Adding GNPs did not enhance efficacy, indicating LNP_DTX–P_ was the main driver under this regimen. These results demonstrate nanocarrier-enabled radiosensitization in vivo and support further studies toward clinical translation.

## 1. Introduction

Cancer is one of the leading causes of death worldwide, accounting for nearly one in six deaths globally [1]. Surgery, chemotherapy, and radiotherapy (RT) form the backbone of cancer treatments, but each faces significant limitations [2]. Surgery is often unfeasible in advanced-stage cancers, chemotherapy lacks tumor specificity and causes systemic toxicity, and RT is constrained by dose-limiting damage to surrounding healthy tissues [2]. Nanotechnology aims to overcome some of the limitations associated with cancer therapies. Nanoparticles (NPs) can passively accumulate in tumors through the enhanced permeability and retention (EPR) effect, a result of a leaky tumor vasculature and poor lymphatic drainage of the tumor microenvironment (TME) [3]. Additionally, NPs can also be actively targeted by attaching ligands that bind to specific receptors on cancer cells, thus enhancing selective uptake and therapeutic efficacy [3].

To improve RT, we utilized two radiosensitizers. The first is gold nanoparticles (GNPs) due to their biocompatibility, low toxicity, versatility, and safety [4]. To enhance tumor selectivity and biological stability, GNPs were functionalized with polyethylene glycol (PEG) to reduce immune clearance [5] and with RGD peptides to facilitate targeted binding to integrin receptors overexpressed on cancer cells [6]. Once localized in the tumor, GNPs amplify radiation effects by increasing local dose deposition via the photoelectric effect and Compton scattering, owing to their high atomic number and greater radiation interaction compared to soft tissues [7]. Beyond GNPs, other radiosensitizers have been explored to improve RT. A similar metallic radiosensitizer, NBTXR3 (hafnium oxide NPs), has progressed to phase 2–3 clinical trials for soft tissue sarcomas and head and neck cancers [8]. Small molecule radiosensitizers (e.g., gemcitabine, nimorazole, and PARP inhibitors) have also been investigated to increase DNA damage or impair repair mechanisms [9,10,11]. However, in this study, we selected docetaxel (DTX) as our second radiosensitizer, as it is a widely used chemotherapeutic agent across multiple cancer types, including breast, lung, and prostate cancers [12]. It acts as a radiosensitizer primarily by interfering with the cell cycle and enhancing radiation-induced DNA damage [13,14]. It stabilizes microtubules (MTs), leading to mitotic arrest in the G2/M phase, one of the most radiosensitive phases of the cell cycle [14]. By trapping cancer cells in this phase, DTX increases their vulnerability to radiation. To improve its solubility, pharmacokinetics, and tumor-specific delivery, DTX was converted into a prodrug and encapsulated within lipid nanoparticles (LNPs) forming LNP_DTX–P_. This formulation improves drug stability, prolongs systemic circulation, and benefits from the established clinical success of multiple FDA-approved LNP-based therapeutics [15,16].

**Figure 1:**
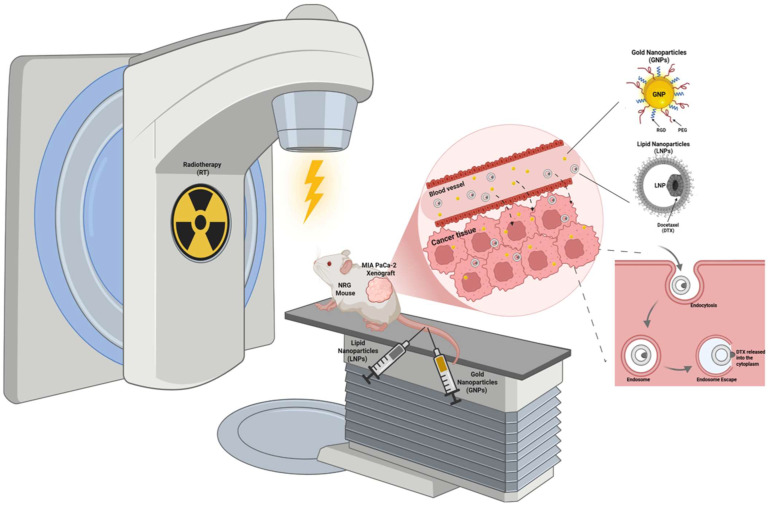
Schematic representation of the in vivo experimental setup. NRG mice bearing subcutaneous MIA PaCa-2 pancreatic cancer xenografts were treated with gold nanoparticles (GNPs) functionalized with polyethylene glycol (PEG) and RGD peptides, lipid nanoparticles encapsulating a docetaxel prodrug (LNP_DTX–P_), and external beam radiotherapy (RT). The combination therapy was designed to evaluate the radiosensitizing effects of GNPs and LNP_DTX–P_ on tumor response. The diagram highlights the enhanced permeability and retention (EPR)-mediated tumor accumulation of NPs. NPs undergo endocytosis by cancer cells, followed by endosomal escape and intracellular release into the cytoplasm. Concurrent RT is applied to the tumor site to synergize with NP-mediated drug delivery and radiosensitization, improving tumor control and survival benefit. Created with BioRender.com.

In our earlier work, we demonstrated radiosensitization in vitro across both 2D monolayers and 3D (monoculture and co-culture) spheroids, where GNPs with DTX or LNP_DTX–P_ plus RT reduced spheroid growth, increased DNA double-strand breaks (DSBs), and lowered cell viability [17,18,19]. In vivo, we showed that the DTX + GNP + RT triple combination suppressed tumor growth and extended median survival in MIA PaCa-2 xenografts compared to conventional RT [17]. We also demonstrated that LNP_DTX–P_ approximately doubled intratumoral GNPs accumulation in the tumor compared with control, and characterized biodistribution across major organs, alongside evidence of cell accumulation in the G2/M phase consistent with DTX effects [18]. However, no study has yet evaluated whether LNP_DTX–P_ in combination with GNPs, can radiosensitize pancreatic tumors in vivo. This represents an important gap because pancreatic cancer is highly resistant to conventional therapy. Therefore, the novelty of the present work lies in providing the first in vivo proof-of-concept that LNP_DTX–P_ improves RT efficacy in pancreatic cancer xenografts, with or without GNPs (Figure 1). Subcutaneous MIA PaCa-2 xenografts were established in NRG mice, which then received treatments of GNPs (2 mg/kg) and LNP_DTX–P_ (6 mg/kg), followed by 5 Gy of external beam RT. Our study directly builds on our prior work and aims to establish a foundation for clinically relevant nanocarrier-based radiosensitization strategies. We hypothesize that LNP_DTX–P_ timed to peak G2/M would improve RT efficacy in vivo and that adding GNPs might provide further benefit.

## 2. Materials and Methods

### 2.1. Synthesis and Characterization of Gold Nanoparticles

Gold nanoparticles (GNPs) were synthesized via the citrate reduction method [20]. Transmission electron microscopy (TEM) was performed using a Hitachi SU9000 (Hitachi High-Tech Corporation, Tokyo, Japan) ultra-high-resolution scanning electron microscope. To enhance their biological stability and targeting capabilities, GNPs were functionalized with polyethylene glycol (PEG, 2000 Da) and arginine-glycine-aspartic acid (RGD, 1600 Da) peptides. Functionalization was performed at a surface ratio of one PEG molecule per nm^2^ and one RGD molecule for every two PEG molecules. PEGylation was intended to reduce aggregation and evade immune recognition, while RGD facilitates integrin-specific binding for tumor targeting.

### 2.2. Synthesis and Characterization of Lipid Nanoparticles

Lipid nanoparticles (LNPs) were synthesized using rapid mixing, as outlined in our previous publication [21]. It involved dissolving DSPC, PEG-DSPE, cholesterol, and DTX prodrug in ethanol, achieving a lipid concentration of 10 mM with a molar ratio of 49:1:40:10. The ethanol solution was then merged with phosphate-buffered saline (PBS) in a 1:4 volume-to-volume ratio at a total flow rate of 40 mL/min. Subsequent steps included dialysis, sterile filtration, and particle size analysis via dynamic light scattering using a Malvern Zetasizer NanoZS (Malvern Instruments, Worcestershire, UK). The LNPs’ cholesterol and phospholipid contents were quantified to determine lipid concentration, utilizing Cholesterol E Assay and Phospholipids C Assay kits (Wako Chemicals, Richmond, VA, USA). The amount of DTX prodrug was measured with ultra-performance liquid chromatography (UPLC), and its encapsulation efficiency in the LNPs was assessed by comparing the ratios of prodrug to cholesterol in the final product against the initial lipid mixture. Cryogenic TEM of the LNP_DTX–P_ was conducted at The University of British Columbia’s High-Resolution Macromolecular Cryo-Electron Microscopy Facility. For the synthesis, DSPC and PEG-DSPE were sourced from Avanti Polar Lipids (Alabaster, AL, USA), cholesterol was obtained from Sigma-Aldrich (St. Louis, MO, USA), and DTX was acquired from eNovation Chemicals (Bridgewater, NJ, USA). Particle size and optical properties were characterized using a Perkin Elmer λ365 UV–Vis spectrophotometer (Waltham, MA, USA), with further assessments of hydrodynamic diameter and ζ-potential performed using a LiteSizer 500 (Anton Paar, Graz, Austria) to evaluate colloidal stability before and after functionalization.

### 2.3. Xenograft Model and Treatment Protocols

Female NOD-*Rag1^null^ IL2rg^null^* (NRG) mice were obtained from the BC Cancer Research Centre Animal Resource Centre (BCCRC ARC) and housed under standard conditions. MIA PaCa-2 (ATCC^®^ CRL-1420™) was sourced in 2021, expanded from a cryopreserved lab stock originally derived from the ATCC vial, tested mycoplasma-negative, and stored in liquid nitrogen until use. For xenograft implantation, cells between passages 3 and 10 with 80–90% confluency were cultured in Dulbecco’s Modified Eagle Medium (DMEM; Gibco, Thermo Fisher Scientific, Waltham, MA, USA) supplemented with 2 mM L-glutamine, 10% fetal bovine serum (FBS), and 2.5% horse serum, maintained at 37 °C in a humidified incubator with 5% CO_2_. On Day 0, each mouse received a subcutaneous injection of 5 × 10^6^ MIA PaCa-2 cells in 100 µL of medium using a 27-gauge needle, as per SOP-AF-018. Tumor volume was monitored biweekly using digital calipers once the tumors reached ~275-300 mm^3^. Tumor volume was calculated using the standard formula: (length × width^2^)/2, with length measured as the longest tumor dimension. Tumors were allowed to grow to a maximum size of 800 mm^3^ unless otherwise dictated by humane endpoints. Treatment began when tumors reached the target size. Mice were randomized into treatment groups of n = 8 animals each and administered intravenous injections of GNPs (2 mg/kg), and LNP_DTX–P_ (6 mg/kg), using a 28-gauge needle depending on their assigned treatment group. RT was delivered as a single 5 Gy dose using clinical linear accelerators at the BC Cancer Clinic (Vancouver). All procedures involving animals were approved by the University of British Columbia Institutional Animal Care Committee (IACC) under service-oriented protocols (#A18-0276 and #A22-0274), in accordance with the Canadian Council on Animal Care Guidelines. The study methodology and related data are available to the IACC upon request and under confidentiality agreement.

### 2.4. Statistical Analysis

Tumor volumes were analyzed with a linear mixed-effects model with fixed effects for group, day, and group × day and a random intercept for mouse to account for repeated measures. When residuals indicated skew or unequal variance, volumes were analyzed on the log scale and back-transformed for presentation. A pre-specified snapshot comparison at the last common time point was performed using ANCOVA adjusting for each mouse’s baseline volume, with Tukey-adjusted pairwise tests. Survival is summarized with Kaplan–Meier curves; we report median survival at selected times. Figures show means with SEM. Group sizes were n = 8 unless otherwise stated. Mice were randomized at enrolment, measurements were recorded blinded, humane endpoints were pre-specified, and any exclusions with reasons are reported in the *Results*.

## 3. Results & Discussion

### 3.1. GNP and LNP_DTX–P_ Characterization

**Figure 2:**
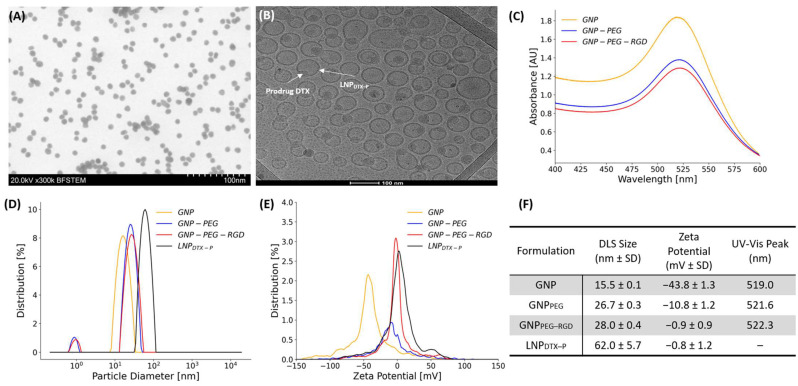
Characterization of GNPs and LNPs, and their functionalized forms. (**A**) BFSTEM image of spherical gold nanoparticles. (**B**) Cryo-TEM image of LNP_DTX–P_. (**C**) UV–Vis absorbance spectra of GNPs before and after PEGylation and RGD surface modification. (**D**) DLS data showing particle size distributions of GNP formulations and LNP_DTX–P_. (**E**) Zeta potential distributions for the various nanoparticle formulations, demonstrating surface charge changes after PEG and RGD modifications and after LNP_DTX–P_ integration. (**F**) Summary table of key physicochemical properties for all nanoparticle formulations including peak absorbance wavelength, hydrodynamic diameter, and zeta potential (mean ± SD, n = 3).

The characterization of GNPs and LNPs is presented in Figure 2, confirming the successful formulation of both NPs. We previously reported the physicochemical characterization and release kinetics of LNP_DTX–P_ [21], establishing its stability and pharmacological profile, which provided the foundation for the current in vivo investigation. Surface modification of GNPs with PEG and RGD was demonstrated by incremental increases in hydrodynamic diameter and stepwise reductions in zeta potential [22], while loading of the docetaxel prodrug (DTX–P) into LNPs was evidenced by imaging. Bright-field scanning transmission electron microscopy (BFSTEM) (Figure 2A) confirmed that citrate-reduced GNPs exhibited a spherical morphology with a relatively narrow size distribution (~15 nm diameter), while cryo-TEM imaging revealed that LNP_DTX–P_ particles were uniformly spherical (~60 nm diameter) with visible encapsulation of the hydrophobic prodrug within the lipid matrix (Figure 2B).

As shown in Figure 2C–F, dynamic light scattering (DLS) analysis further validated these observations, with unmodified GNPs measuring 15.5 ± 0.1 nm in hydrodynamic diameter. PEGylation increased the size to 26.7 ± 0.3 nm, and subsequent RGD conjugation yielded particles of 28.0 ± 0.4 nm. LNP_DTX–P_ particles, in contrast, exhibited a significantly larger size of 62.0 ± 5.7 nm, which is suitable for EPR-mediated tumor targeting [23]. Zeta potential measurements demonstrated a highly negative surface charge for bare GNPs (−43.8 ± 1.3 mV), which was reduced to −10.8 ± 1.2 mV after PEGylation and nearly neutralized (−0.9 ± 0.9 mV) following RGD conjugation. Similarly, LNP_DTX–P_ exhibited near-neutral zeta potential (−0.8 ± 1.2 mV), an important feature for minimizing opsonization and prolonging systemic circulation [24]. UV–Vis spectroscopy revealed a surface plasmon resonance peak at 519.0 nm for unmodified GNPs, which red-shifted to 521.6 nm and 522.3 nm upon PEG and RGD modification, respectively, consistent with surface conjugation altering the local dielectric environment [25]. As expected, LNP_DTX–P_ showed no SPR peak due to its lipid-based nature [26]. Both GNPs and LNP_DTX–P_ demonstrated physicochemical stability over several days under refrigerated conditions, maintaining consistent size, surface charge, and dispersion profiles. These results confirm the successful synthesis, surface conjugation, stability, and functional loading of GNPs and LNPs. The precise control over physicochemical properties including size, charge, and surface functionality enables favorable biodistribution, tumor accumulation, and cellular uptake in our in vivo experiments [27].

### 3.2. Summary of Prior GNPs + LNP_DTX–P_ + RT Data In Vitro and In Vivo

Previously, we investigated how LNP_DTX–P_ affects intratumoral GNP uptake and the cell cycle in vivo. Using MIA PaCa-2 human pancreatic cancer cells implanted subcutaneously in NRG mice, we administered clinically applicable intravenous doses of 2 mg/kg GNPs and 6 mg/kg LNP_DTX–P_ and quantified gold content in tumors over time while analyzing cell-cycle distributions [18]. Quantitative analyses showed that tumors treated with LNP_DTX–P_ plus GNPs exhibited significantly higher GNP accumulation than those treated with GNPs alone, an increase comparable to that seen with free DTX at equivalent doses, as previously reported [17,18]. This elevated accumulation is largely attributable to DTX effects on microtubules (MTs), which are essential for intracellular transport and cell division. GNPs primarily enter cells via receptor-mediated endocytosis, a process that does not rely on MTs [28], but once internalized, endocytic vesicles traffic along MTs within the cell [29]. DTX, an antimitotic agent, disrupts MT dynamics and arrests cells in the radiosensitive G2/M phase [29]. These actions increase intracellular GNP burden through two mechanisms: first, G2/M arrest prevents dilution of GNPs during mitosis; second, MT disruption impairs intracellular trafficking and exocytosis, prolonging intracellular retention.

Flow cytometry previously demonstrated that LNP_DTX–P_ effectively induces G2/M arrest in vivo, consistent with our in vitro findings and validating its function as an MT-targeting formulation [18]. Together, these data highlight LNP_DTX–P_ as an effective alternative to conventional DTX and support its use in combination regimens, particularly where synchronized radiosensitization during G2/M and improved GNP retention are advantageous [17,18]. Thus, these findings justify further investigation of combining GNPs, LNP_DTX–P_, and RT. Building on this foundation, we now assess whether adding RT to LNP_DTX–P_ and GNPs improves in vivo outcomes, using a single-fraction protocol timed to the G2/M window.

### 3.3. In Vivo Tumor Suppression and Survival Outcomes Following GNPs + LNP_DTX–P_ + RT

**Figure 3:**
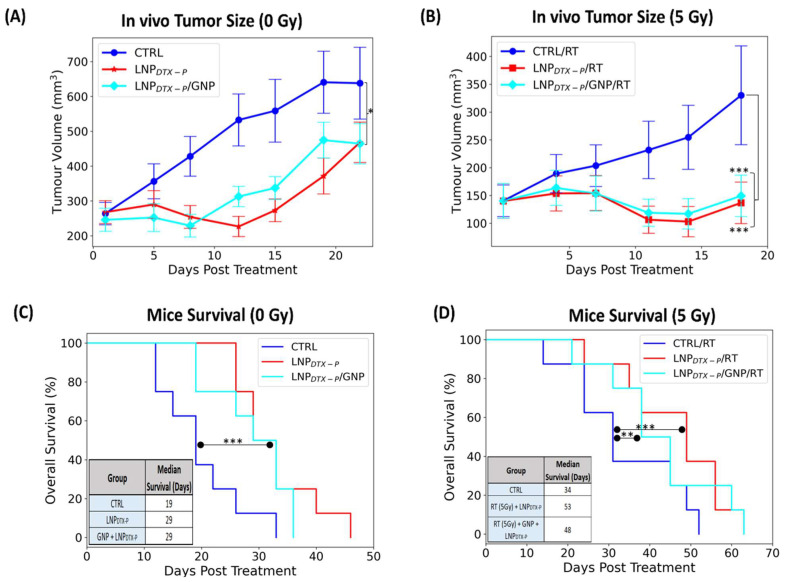
In vivo evaluation of treatment efficacy in NRG mice bearing MIA PaCa-2 pancreatic tumor xenografts following injection with GNPs and LNP_DTX–P_, with and without RT. (**A**) Tumor growth curves (0 Gy) over 21 days post-treatment show partial suppression in the LNP_DTX–P_ + GNP and LNP_DTX–P_ groups compared to controls. (**B**) Tumor volumes following 5 Gy RT reveal significantly enhanced tumor growth inhibition when GNPs were combined with LNP_DTX–P_. (**C**) Kaplan–Meier survival curves and median survival data for untreated mice or those receiving drug treatments without RT. (**D**) Survival curves and median survival following 5 Gy RT demonstrate a clear survival benefit with LNP_DTX–P_ therapies compared to controls. The inset summarizing median survival (in days) across all groups. Error bars represent SEM (n = 8 per group). Statistical significance is denoted as * for *p* < 0.05, ** for *p* < 0.01, and *** for *p* < 0.001.

A single 5 Gy dose of RT was delivered using a 6 MV clinical linear accelerator 24 h after GNP and LNP_DTX–P_ injection. This dose was selected because 2 Gy, the standard clinical fraction, is generally insufficient to produce measurable tumor effects in vivo, whereas higher doses (8–10 Gy) can cause near-complete regression and mask the NPs radiosensitization effects [7]. The 24 h timepoint was chosen because it coincided with peak G2/M cell-cycle arrest [18]. Tumor growth was tracked until mice reached a predetermined endpoint of 800 mm^3^. Tumor volume data (Figure 3A,B) show distinct outcomes in non-irradiated (0 Gy) and irradiated (5 Gy) groups. Without RT, tumor progression was fastest in the control group, while treatment with LNP_DTX–P_ significantly slowed tumor growth (~17% reduction, *p* < 0.05), an effect not seen with free DTX [17]. The slowed tumor growth mediated by LNP_DTX–P_ is attributed to the improved LNP accumulation in the tumor [30]. Upon applying 5 Gy RT, treatment efficacy improved across all groups (Figure 3B). The most pronounced tumor control occurred in mice receiving LNP_DTX–P_ alone or in combination with GNPs, where tumor volumes remained largely stable over 10–15 days. These groups suppressed tumor growth by nearly 40% compared to controls by Day 20, consistent with enhanced delivery and retention of the liposomal LNP formulation. Surprisingly, the triple combination (GNP + LNP_DTX–P_ + RT) did not significantly outperform LNP_DTX–P_ + RT alone. This finding is partially consistent with our previous studies, where GNP radiosensitization was modest on its own but became evident when combined with free DTX due to their synergistic interaction [17]. However, in the present study LNP_DTX–P_ outperformed free DTX both with and without GNPs, achieving greater tumor control (~20% reduction, *p* < 0.001) [17] and reinforcing the pharmacological advantage of LNP_DTX–P_ [30]. This suggests that the potent activity of the LNP formulation may have overshadowed both the additive radiosensitization impact of GNPs and their potential synergy with DTX, which represents a limitation of the current study and warrants further mechanistic investigation.

Kaplan–Meier survival curves (Figure 3C,D) provide further insight into treatment benefits. Without RT, LNP_DTX–P_ and LNP_DTX–P_ + GNP treatments extended median survival to 29 days (*p* < 0.001), compared to 19 days in the control group, again outperforming free DTX and DTX + GNP and aligning with tumor volume trends (Figure 3C). When combined with RT, survival improved across all groups [17], with the LNP_DTX–P_ + RT group showing the highest median survival of 53 days (*p* < 0.01), followed closely by the LNP_DTX–P_ + GNP + RT group at 48 days (*p* < 0.001) (Figure 3D). These significantly outperformed controls (34 days), GNP (38 days), free DTX (34.5 days), and free DTX + GNP (44.5 days), confirming the sustained therapeutic benefit of LNP_DTX–P_ [17]. Remarkably, achieving such tumor growth inhibition with a single administration is uncommon, as most preclinical and clinical regimens require multiple doses to achieve comparable effects. This suggests that incorporating a more clinically realistic or intensified dosing schedule could further improve tumor control and survival benefit, potentially achieving not only slowed growth but even tumor regression. These findings highlight the importance of LNPs as a drug delivery system, offering more effective tumor control and survival benefit compared to conventional drugs, even at a lower dose. It is also important to note that systemic toxicity for the same dosing regimens of free DTX and LNP_DTX–P_ was previously assessed [18], where body weight monitoring, biodistribution, and organ histology (H&E of liver, kidney, and spleen) showed no observable damage at the tested doses. While the present manuscript focuses on proof-of-concept efficacy, future work will require dedicated toxicity and pharmacokinetic studies to fully support clinical translation.

## 4. Conclusions

This study shows that combining LNP_DTX–P_ + RT significantly improved tumor control and survival in vivo. In a single-dose regimen timed to G2/M, LNP_DTX–P_ with 5 Gy RT produced greater tumor suppression and prolonged survival compared with controls and free DTX ± GNPs. Taken together, these results suggest that LNP_DTX–P_ delivery improves therapeutic efficacy in vivo compared to free DTX and RT, supporting the potential for dose-sparing regimens. Such dose-sparing strategies could in turn reduce systemic toxicity, although this remains speculative and will require confirmation in dedicated safety studies. Future work should assess fractionated RT and repeated dosing, pharmacokinetics and long-term toxicity, and performance in orthotopic or immunocompetent models to support clinical translation. If validated clinically, this approach could improve outcomes in difficult-to-treat cancers such as pancreatic carcinoma.

## Data Availability

The datasets generated and/or analyzed during this study are available from the corresponding author upon reasonable request.

## Abbreviations

The following abbreviations are used in this manuscript:

RT	Radiotherapy
TME	Tumor Microenvironment
NPs	Nanoparticles
GNPs	Gold Nanoparticles
PEG	Polyethylene Glycol
RGD	Arginine-Glycine-Aspartic Acid
LNPs	Lipid Nanoparticles
DTX	Docetaxel
EPR	Enhanced Permeability and Retention
LNP_DTX–P_	Lipid Nanoparticles Encapsulating Docetaxel Prodrug
TEM	Transmission Electron Microscopy
BFSTEM	Bright-Field Scanning Transmission Electron Microscopy
MTs	Microtubules
PBS	Phosphate-Buffered Saline
UPLC	Ultra-Performance Liquid Chromatography
DSPC	1,2-Distearoyl-sn-Glycero-3-Phosphocholine
PEG-DSPE	Polyethylene Glycol-Distearoyl Phosphatidylethanolamine
DMEM	Dulbecco’s Modified Eagle’s Medium
NRG	NOD-*Rag1^null^ IL2rg^null^* (immunodeficient mice)
FBS	Fetal Bovine Serum
RES	Reticuloendothelial System
DLS	Dynamic light scattering
DSBs	Double-Strand Breaks
